# Cross-country multi-modal evidence links *Aspergillus* to biliary atresia

**DOI:** 10.1186/s13099-025-00772-7

**Published:** 2025-11-17

**Authors:** Song-Wei Huang, Chia-Ray Lin, Ya-Hui Chang, Yen-Hsuan Ni, Huey-Ling Chen, Hong-Hsing Liu

**Affiliations:** 1 Institute of Molecular and Genomic Medicine, National Health Research Institutes, Zhunan Township, Miaoli County, 35053 Taiwan; 2 Pediatrics Department, National Taiwan University Children’s Hospital, Taipei, 100225 Taiwan; 3 Pediatrics, En Chu Kong Hospital, New Taipei City, 237414 Taiwan

**Keywords:** Clay-colored stool, Hyperbilirubinemia, Kasai portoenterostomy, Aspergillosis, Fungal colonization

## Abstract

**Background:**

Biliary atresia (BA) is the leading cause of pediatric liver transplantation. It is characterized by progressive extrahepatic bile duct obstruction in young infants. Inspired by the success of antifungal treatment in a newborn with BA-related obstructive cholangitis, we explored a potential link between BA and fungi, particularly *Aspergillus*. Fecal DNA was analyzed using 18S ribosomal sequencing and validated with a published fecal metagenomic dataset. Epidemiological data from the UK, Taiwan, and Japan were also examined.

**Results:**

Gut *Aspergillus* was exclusively detected in BA cases, suggesting it may be a potential trigger. Independent fecal metagenomic data from China and epidemiological correlations further supported this hypothesis. In the UK, BA presentations strongly correlated (*r* = 0.98, 95% CI [0.36, 1.0], *p* = 0.02) with *Aspergillosis*, but not with *Candidiasis*, during the COVID-19 lockdown. In Taiwan, a decade of data showed BA incidence was significantly associated (*r* = 0.78, 95% CI [0.29, 0.94], *p* = 0.01) with yearly *Aspergillus*-positive isolates among cancer-adjusted hospital admissions. In Japan, BA cases over 25 years correlated significantly (*r* = 0.85, 95% CI [0.37, 0.97], *p* = 0.01) with visceral *Aspergillus* burdens in autopsied cases, but not with other fungal infections.

**Conclusions:**

The resolution of obstructive cholangitis in the antifungal-treated index case, together with multi-modal, cross-country evidence, highlights a potential link between gut *Aspergillus* and BA. Although limited by small sample size, retrospective design, and lack of mechanistic validation, the study may still be interpreted as hypothesis-generating and underscores the need for prospective studies to validate and extend these observations.

**Supplementary Information:**

The online version contains supplementary material available at 10.1186/s13099-025-00772-7.

## Introduction

Biliary atresia (BA) is a disease characterized by extrahepatic bile duct obliteration in young infants. It occurs either as an isolated form or as part of a syndrome, sometimes accompanied by laterality defects [1]. The syndromic form is far less common, and this study primarily focuses on the isolated form. Affected infants are typically asymptomatic at birth but develop signs such as prolonged jaundice and acholic stool between two and six weeks of age. Kasai portoenterostomy, which reroutes bile flow from the intrahepatic ducts to the intestine, remains the standard first-line treatment for BA [2]. Nevertheless, up to two-thirds of patients eventually require liver transplantation due to progressive bile duct obstruction and end-stage liver cirrhosis [3].

Multiple genetic factors have been linked to BA, including genes related to ciliopathy and hepatobiliary development, as well as those involved in inflammation and fibrogenesis [4]. A distinct BA variant has recently been identified in patients with null mutations of *GSTM1*, resulting in impaired detoxification of fungal aflatoxins [5]. Viral pathogens [6, 7] and plant toxins [8] have also been proposed as potential triggers. In summary, BA development likely involves multiple factors, potentially including environmental triggers and subsequent immune dysregulation in susceptible infants. Here, we provide evidence that gut *Aspergillus* spp. may serve as a distinctive trigger for BA, based on sequences identified in our cases and in an independent dataset of fecal shotgun metagenomes, where *Aspergillus* showed the largest mean difference between BA and controls among fungi with significant differences. Furthermore, correlations between BA occurrences and the clinical burdens of *Aspergillus* spp. observed in the UK, Taiwan, and Japan further support this hypothesis.

## Methods

### Aim, design, and setting of the study

Prompted by a clinical case in which antifungal therapy improved neonatal cholangitis, we investigated a potential association between gut fungal colonization, particularly by *Aspergillus* spp., and the development of BA in infants. We employed a comprehensive approach that integrated clinical case reviews, molecular sequencing, and validation using a publicly available fecal metagenomic dataset. Clinical samples were analyzed from two hospitals in Taiwan: National Taiwan University Children’s Hospital and En Chu Kong Hospital. Participant demographics and diagnostic information are summarized in Table 1. To further support our findings and assess environmental fungal exposure, we performed epidemiological analyses across Taiwan, the United Kingdom, and Japan.

**Table 1:** BLAST hits of nested 18S V7–V8 or V4–V8 sequences

Sample^^^	Age(mo)	Diagnosis	V7–V8 hits^*^	V4–V8 hits^*^	Fungi^†^
D28	0.9	Cholangitis	*Aspergillus* spp. *Aspergillus* spp.	*Penicillium limosum* *Penicillium* spp.	U
D31	1.0	Cholangitis	No PCR bands	No PCR bands	N
D38	1.3	Cholangitis	No PCR bands	*Gorilla gorilla* *Pan troglodytes*	N
BA01	1.7	Biliary Atresia	*Cerrena* spp. *Cerrena* spp.	*Cerrena* spp. *Cerrena* spp.	U
BA02	1.1	Biliary Atresia	No PCR bands	No PCR bands	N
BA03	2.4	Biliary Atresia	Uncultured fungus *Candida parapsilosis*	*Candida parapsilosis* *Candida metapsilosis*	C
BA04	3.9	Biliary Atresia	No PCR bands	*Homo sapiens* *Gorilla gorilla*	N
BA05	0.6	Biliary Atresia	*Aspergillus* spp. *Aspergillus* spp.	*Aspergillus restrictus* *Aspergillus cristatus*	U
BA06	0.7	Biliary Atresia	PCR bands not cloned	*Hydnochaete duportii(4)* *Phellinidium pouzarii(3)* Uncultured fungus*(3)* *Aspergillus penicillioides(2)* *Aspergillus flavus(1)* *Fungal* spp.*(1)* *Trichaptum abietinum(1)* *Skvortzovia pinicola(1)*	U
BA07	1.3	Biliary Atresia	No PCR bands	No PCR bands	N
BA08	1.4	Biliary Atresia	No PCR bands	No PCR bands	N
BA09	2.2	Biliary Atresia	No PCR bands	No PCR bands	N
BA10	0.5	Biliary Atresia	No PCR bands	No PCR bands	N
Control01	1.0	Normal	No PCR bands	No PCR bands	N
Control02	1.0	Normal	No PCR bands	No PCR bands	N
Control03	2.0	Normal	Uncultured fungus *Candida parapsilosis*	*Candida parapsilosis* *Candida metapsilosis*	C
Control04	2.0	Normal	No PCR bands	No PCR bands	N
Control05	1.0	Normal	*Candida albicans* *Candida albicans*	*Candida albicans* *Candida albicans*	C
Control06	2.0	Normal	*Candida albicans* *Candida albicans*	*Candida albicans* *Candida albicans*	C
Control07	1.0	Normal	Uncultured fungus *Candida parapsilosis*	*Candida parapsilosis* *Candida metapsilosis*	C
Control08	2.0	Normal	*Malassezia furfur* *Malassezia furfur*	*Malassezia furfur* *Malassezia furfur*	C
Control09	2.0	5β-Reductase Deficiency	Uncultured fungus Uncultured fungus	*Gorilla gorilla* *Kogia breviceps*	N
Control10	1.1	Disorder of Bilirubin Metabolism	No PCR bands	No PCR bands	N
Control11	1.6	CMV^‡^ Infection	No PCR bands	*Pan paniscus* *Pan troglodytes*	N
Control12	2.3	NICCD^‡^	Uncultured fungus *Malassezia arunalokei*	Uncultured fungus *Selenicereus undatus*	C
Control13	2.7	Choledochal Cyst	Uncultured* Malassezia* *Malassezia pachydermatis*	*Pan troglodytes* *Pan troglodytes*	C

^^^BA samples were collected prior to Kasai portoenterostomy.

^*^First and second hits against the NCBI nr/nt database are presented. For BA06, results are based on eight V4–V8 PCR clones, with hit counts shown in parentheses.

^†^Fungal status designations for each sample: U = non-commensal fungi, C = commensal fungi, N = no PCR bands.

^‡^CMV, cytomegalovirus; NICCD, neonatal intrahepatic cholestasis caused by citrin deficiency.

### Molecular biology experiments

Detailed protocols for fecal DNA extraction, 16S/18S PCR, Sanger sequencing, and TA cloning are provided in the Supplementary Methods (Additional file 1).

### Bioinformatic analyses

Sanger sequencing results were processed in SnapGene (Dotmatics, Boston, MA, USA; www.snapgene.com) for visualization and editing. Taxonomic identities were assigned through nucleotide BLAST [9] searches against the NCBI nr/nt database [10] using the NCBI web server. Data visualization was performed in the Python library seaborn [11]. For fungal taxonomic profiling, Kraken 2 [12] was employed. From each sample, 2 million sequence reads were randomly selected, and analyses were repeated five times. The averaged output from these replicates, totaling 10 million reads, was used to represent each sample. Forward and reverse reads were processed separately to ensure consistency and coverage.

### Estimations of fungal burdens from published data

To estimate case numbers from published figures, Fig. 1 in Sung et al. [13] was digitally enlarged by approximately 330%, establishing a visual scale in which 5 cm represented 300 cases of *Aspergillosis* and 4 cm represented 100 cases of *Candidiasis*. The vertical heights of plotted data points, corresponding to monthly data from March to June across 2018–2021, were measured manually with a ruler. Similarly, incidence estimates for *Aspergillus* spp. were derived from Fig. 1 in Hsiue et al. [14], where 1 mm equaled 1 case per 1,000 hospital admissions and 5.3 cm represented 20% of total cancer admissions. To ensure reproducibility and minimize measurement bias, manual measurements were independently repeated by five additional individuals, including three non-authors, each using randomly adjusted image scales. Correlation coefficients showed excellent consistency across raters: UK-*Aspergillus* (mean *r* = 0.985, standard deviation [SD] = 0.002, coefficient of variation [CV] = 0.2%), UK-*Candida* (mean *r* = 0.55, SD = 0.028, CV = 5.2%), and TW-*Aspergillus* (mean *r* = 0.77, SD = 0.019, CV = 2.5%). Here, SD represents the dispersion of values around the mean, and CV (SD ÷ mean × 100%) indicates relative variability. These results demonstrate that the manual measurement approach is highly reproducible (Fig 3 and Figure S2, Additional file 1). For Japan, incidence data were extracted from published tables by Kume et al. [15] and Suzuki et al. [16], and directly retrieved from the Japanese Biliary Atresia Registry (https://jbas.net/registration-historicaldata).

**Fig. 1:**
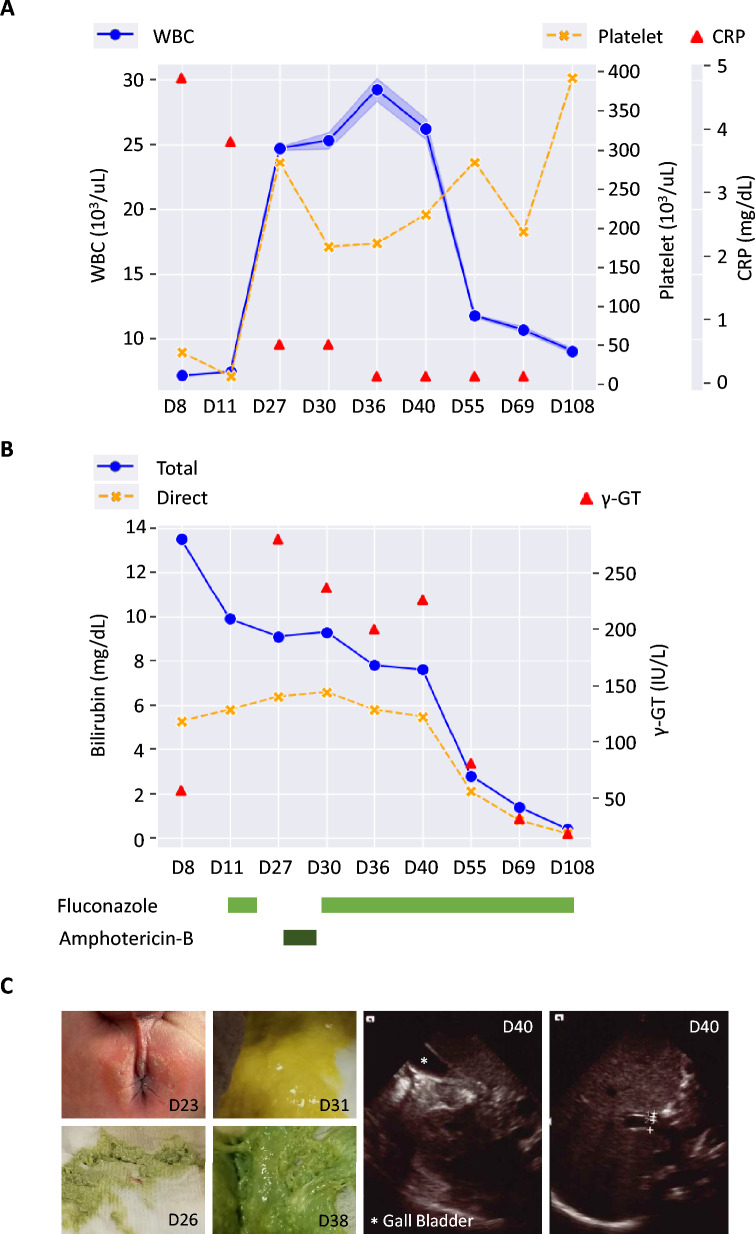
Clinical presentation of the index cholangitis case. (**A**) Platelet and WBC profiles differed between the early gastroenteritis phase (D8–D11) and the later cholangitis phase (D27–D40), with high and low C-reactive protein (CRP) values, respectively. Shaded areas along WBC trajectories indicate eosinophil counts. (**B**) Blood bilirubin and γ-GT levels declined following fluconazole treatment. (**C**) Perianal dermatophytosis initially coincided with acholic stool. Sequential stool phenotypes and abdominal ultrasonography demonstrated resolution of bile duct obstruction

### Statistics

Statistical analyses were performed to assess associations and differences between groups. Fisher’s exact test was used to evaluate the presence of non-commensal fungi uniquely detected in cases compared with controls. Correlation analyses were conducted using the pearsonr function in SciPy [17], with Pearson’s *r*, 95% confidence intervals, and *p*-values reported. Regression plots display analytically calculated 95% confidence bands around the fitted line. Differences in fungal genus abundance between groups were assessed using Student’s t-test (two-sample, equal variances), with mean differences (Δ), 95% confidence intervals, and *p*-values reported. A two-sided *p* < 0.05 was considered statistically significant. All analyses and visualizations were performed in Python using NumPy, SciPy, pandas, seaborn, and matplotlib.

No a priori sample size or power calculations were performed due to the opportunistic, retrospective design. To guide prospective validation, we estimated required sample sizes from both observed and conservative effect sizes. For prevalence analyses of non-commensal fungi, planning around an absolute difference of 20–30% between BA and controls suggested that approximately 12–19 participants per group would be sufficient at 80% power and α = 0.05 (two-sample proportion test, assuming near-zero prevalence in controls). On a more conservative basis, allowing for a small background prevalence in controls and other planning safeguards, larger samples of approximately 22–36 per group may be advisable. For correlation analyses of annual BA incidence against *Aspergillus* burden, the observed effects (*r* ≈ 0.78–0.98 across countries) implied that ~8–11 annual observations would be sufficient for 80% power. Using a conservative target based on two standard deviations below the cross-country mean (*r* ≈ 0.67) required ~16 observations, while planning for a more moderate correlation (*r* ≈ 0.5) would necessitate ~30 observations. These estimates are intended to guide prospective confirmation and do not alter the exploratory nature of the present work.

## Results

### Fungicidal treatment for neonatal cholangitis

A male newborn was delivered *via* cesarean section at 39 weeks of gestation due to fetal distress. The infant experienced cardiac arrest and exhibited thick meconium staining at birth but was successfully resuscitated and transferred to the Neonatal Intensive Care Unit. Severe hypoglycemia (blood glucose < 2 mg/dL) was detected on admission. Continuous infusion of 10% glucose *via* peripheral lines only barely maintained blood glucose levels. Because central lines were unavailable, early oral feeding with 50% concentrated glucose solution was initiated on day 1 (D1) and continued for one week. Insulin and cortisol responses were within normal ranges on D3, and glucose levels were mostly maintained above 40 mg/dL thereafter, with exceptions on D8 (14 mg/dL) and D25 (39 mg/dL). No metabolic acidosis was observed. Alanine aminotransferase was elevated on D1 (389 IU/L) but returned to near-normal levels by D8 (44 IU/L). The patient’s vital signs remained stable, and no neurological complications were noted.

However, ileus developed later in spite of full antibiotic coverage. Severe thrombocytopenia was observed on D8 and D11, while white blood cell (WBC) counts remained normal (Fig. 1A). A fungal infection was suspected, and intravenous (IV) fluconazole was initiated on D11. Clinical improvement was immediate, and fluconazole was discontinued on D19. However, leukocytosis developed (20,400/μL on D15; 28,300/μL on D19), prompting another course of IV antibiotics (D19–D26), which did not improve clinical parameters (D27, Fig. 1A).

During this period, eosinophilia (D27–D30; shades of WBC, Fig. 1A) and direct hyperbilirubinemia with elevated gamma-glutamyltransferase (γ-GT) were noted (D27, Fig. 1B). The patient also developed perianal dermatophytosis that responded to topical clotrimazole, along with the presence of light-colored stool (D26, Fig. 1C). Suspecting fungal cholangitis, IV amphotericin B was given from D27 to D29 (Fig. 1B). Because bilirubin levels did not improve, antifungal therapy was switched to oral fluconazole on D30 (Fig. 1B). Interestingly, stool color normalized the following day (D31, Fig. 1C), with sustained improvement thereafter (D38, Fig. 1C). Hyperbilirubinemia gradually resolved (Fig. 1B), and abdominal ultrasound revealed a well-distended gallbladder without bile duct obstruction (D40, Fig. 1C).

The patient was discharged on D41 while continuing oral fluconazole. Follow-up monitoring of blood counts, bilirubin, and γ-GT values remained normal (D55–D108, Fig. 1A–B). Oral fluconazole was tapered and discontinued. At two and a half years of age, the most recent evaluation showed that the child had normal growth and development.

### Non-commensal fungi in the bowel

Fungal cultures of blood and stool samples collected during hospitalization were all negative. We used fungal ribosomal 18S PCR [18, 19] to assess suspected fungal colonization from fecal DNA, with bacterial 16S V1–V9 PCR [19] as an internal control (Figure S1A, Additional File 1). A clear 18S V1–V8 band was amplified from the D28 fecal sample (Figure S1A, Additional File 1), despite IV amphotericin B administration from D27 to D29 (Fig. 1B). This V1–V8 band was no longer detectable on D31 and D38 following the start of oral fluconazole on D30 (Fig. 1B). PCR products from the V1–V8 region were further amplified for V7–V8 taxonomic classification by semi-nested PCR (red arrow, Figure S1A, Additional File 1). Sequencing identified *Aspergillus* spp. by nucleotide BLAST against the nr/nt database (Table 1; Figure S1B, Additional File 1).

*Aspergillus* spp. is not considered a typical commensal fungus of the gastrointestinal tract [20]. We hypothesized that BA, a disease often preceded by obstructive cholangitis, might also be associated with colonization by unusual fungi in the bowel. To test this, we examined archived fecal DNA from five BA cases and four healthy controls using 18S V1–V8 and semi-nested V7–V8 PCR (Figure S1C, Additional File 1). Four samples (BA01, BA03, BA05, and Control03) yielded positive results (Table 1; Figure S1C, red arrow, Additional File 1). Sequences from BA01 mapped to *Cerrena* spp. (Figure S1D, Additional File 1), while *Aspergillus* spp. was also identified in BA05. Control03 and BA03 were associated with *Candida*. Because both the cholangitis case and BA05 were linked to bowel colonization by *Aspergillus* spp., we aligned their 18S V7–V8 sequences. At least two loci showed distinct T *vs.* C nucleotide polymorphisms (red arrow, Figure S1E, Additional File 1), suggesting that the two patients were colonized by different *Aspergillus* species.

We expanded our investigation of fecal 18S sequences using semi-nested V4–V8 PCR. Five additional BA samples (BA06–BA10, Table 1), four normal controls (Control05–Control08, Table 1), and five cholestatic disease controls (Control09–Control13, Table 1) were included. Due to sequence heterogeneity in BA06, PCR products were cloned before Sanger sequencing. BLAST analysis revealed multiple fungi in BA06, including *Aspergillus* spp. (BA06, Table 1). Using the longer sequence span from V4–V8 PCR, the D28 sample from the index cholangitis patient mapped to *Penicillium* spp. (D28, Table 1), which is taxonomically related to *Aspergillus* spp. [21]. Among the four additional normal controls and five cholestatic controls, three yielded sequences from *Candida* and three from *Malassezia* (Control05–Control13, Table 1). In summary, three of ten BA samples contained non-commensal fungi, whereas none of the controls did (Table 1). Patients with BA or cholangitis were therefore significantly more likely to harbor non-commensal fungi than controls (*Fisher’s exact test, p* = 0.0311), with *Aspergillus* spp. detected repeatedly.

### Confirmation of *Aspergillus* spp. in BA using public shotgun metagenomes

Building on these findings, we anticipated an expansion of *Aspergillus* populations, particularly in older BA cases. To validate this, we analyzed a publicly available dataset of fecal shotgun metagenomes from Song et al. [22], which revealed an unusual enrichment of *Aspergillus* in BA. The dataset comprised 16 BA cases and 10 matched controls, with half of the BA patients having undergone Kasai portoenterostomy. For each sample, two million forward reads were randomly selected for fungal taxonomic classification using Kraken 2 [12]. Five replicate subsamples were analyzed, totaling ten million reads per sample, and the average genus-level fungal abundance was used for comparisons.

**Fig. 2:**
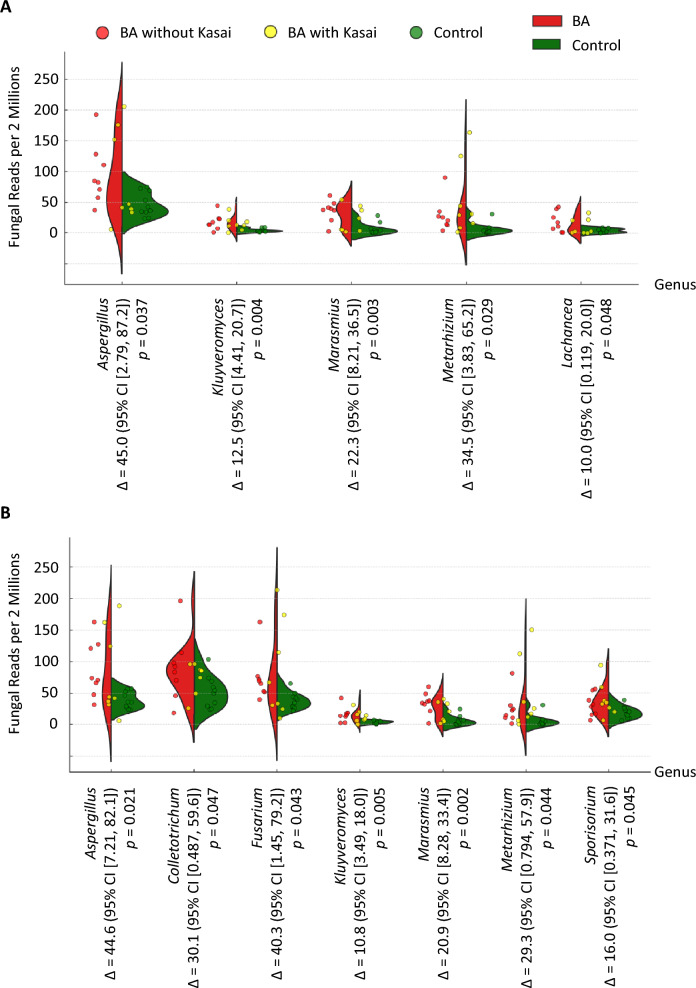
Fungal genera significantly enriched in BA. (**A**) Forward-read analysis. Each point represents the average of five replicates, each derived from two million randomly selected fecal shotgun metagenome reads. Jittered points are overlaid on violin plots for BA and control groups. Only genera with significantly higher abundance in BA are shown; *Aspergillus* spp. exhibited the largest mean difference (Δ). (**B**) Reverse-read analysis. A similar approach identified four overlapping genera (*Aspergillus*, *Kluyveromyces*, *Marasmius*, and *Metarhizium*). In both orientations, *Aspergillus* spp. showed the largest mean difference, with comparable distribution profiles. For all comparisons, mean differences (Δ) and 95% confidence intervals are reported

In the forward reads, total fungal counts were comparable (*p* = 0.672) between BA (3543.5 ± 828.7 per two million reads, standard error [SE]) and controls (2983.6 ± 987.3 per two million reads, SE). Five genera were significantly more abundant in BA: *Aspergillus*, *Kluyveromyces*, *Marasmius*, *Metarhizium*, and *Lachancea* (Fig. 2A). In the reverse reads, four of these genera overlapped (*Aspergillus*, *Kluyveromyces*, *Marasmius*, and *Metarhizium*) (Fig. 2B). In both analyses, *Aspergillus* showed the largest mean difference between BA and controls, with nearly identical abundance distributions across read directions. For each comparison, mean differences (Δ) and 95% confidence intervals are displayed in the figure.

### Significant correlations to the environmental burden of *Aspergillus* spp.

The exclusive detection of *Aspergillus* spp. in BA and cholangitis cases (Table 1), together with confirmation from an independent dataset (Fig 2), led us to hypothesize a potential role for this fungus as a trigger in BA development. A recent UK report indicated that BA presentations declined during COVID-19 lockdowns [23], while another reported a simultaneous decrease in hospitalized patients with aspergillosis [13]. This parallel observation suggests that *Aspergillus* spp. may represent a shared environmental trigger for both conditions. Accordingly, a strong correlation between BA incidence and hospitalizations for aspergillosis would be anticipated.

**Fig. 3:**
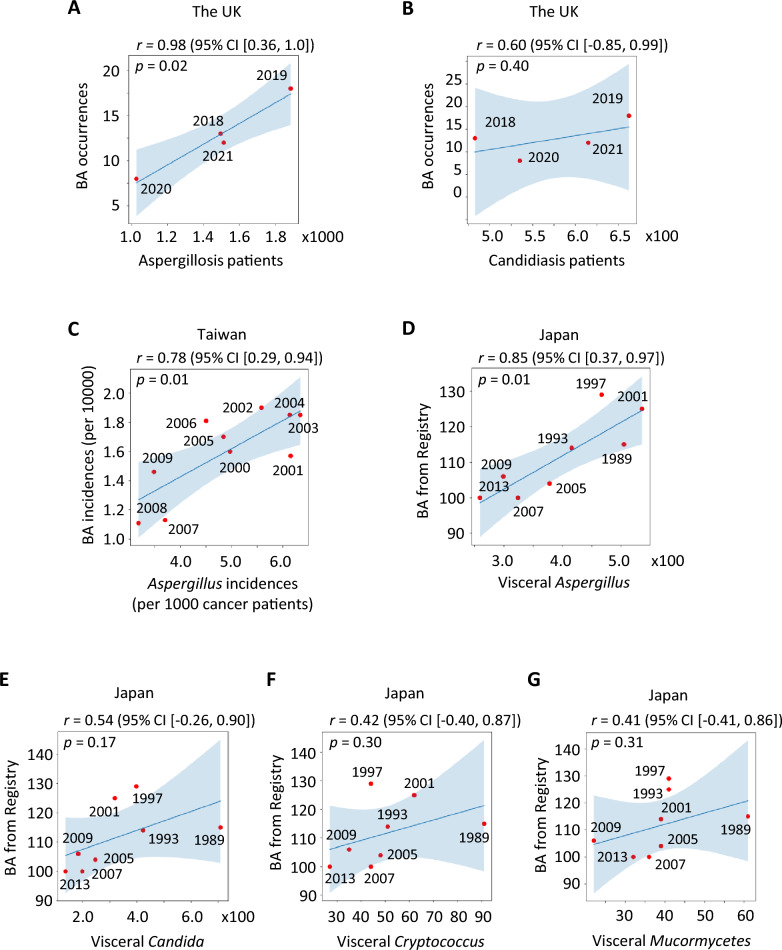
Correlations between BA incidence and environmental burdens of *Aspergillus* spp. (**A–B**) January–June BA presentations (Arshad et al.) were compared with estimated patient numbers from Sung et al. during March–June of 2018–2021. Significant correlation was observed with *Aspergillosis* (*r* = 0.98, 95% CI [0.36, 1.0], *p* = 0.02) but not with *Candidiasis* (*r* = 0.60, 95% CI [–0.85, 0.99], *p* = 0.40). (**C**) In Taiwan, BA incidence from 2000–2009 paralleled *Aspergillus*-positive hospital isolates adjusted for cancer admissions (*r* = 0.78, 95% CI [0.29, 0.94], *p* = 0.01). (**D–G**) In Japan, BA cases reported by the national registry over 25 years strongly correlated with visceral *Aspergillosis* in autopsy cases (*r* = 0.85, 95% CI [0.37, 0.97], *p* = 0.01), but not with visceral *Candidiasis* (*r* = 0.54, 95% CI [–0.26, 0.90], *p* = 0.17), *Cryptococcus* (*r* = 0.42, 95% CI [–0.40, 0.87], *p* = 0.30), or *Mucormycetes* (*r* = 0.41, 95% CI [–0.41, 0.86], *p* = 0.31). For all correlations, shaded regions indicate 95% confidence bands around regression lines

The first UK lockdown lasted from March to June 2020, prompting us to analyze patient numbers during the corresponding months of 2018–2021. Using data reported by Sung et al. [13], we estimated patient numbers for *Aspergillosis* and *Candidiasis* during these periods and compared them with January–June BA case reports from Arshad et al. [23]. Our analysis revealed a strong correlation between BA cases and hospitalizations for *Aspergillosis* (*r* = 0.98, *p* = 0.02; Fig. 3A), supporting our hypothesis of a shared environmental trigger. In contrast, no significant correlation was observed with *Candidiasis* (*r* = 0.60, *p* = 0.40; Fig. 3B).

In Taiwan, the annual incidence of BA from 2000 to 2009 [24, 25] closely paralleled the annual incidence of *Aspergillus*-positive isolates among hospital admissions adjusted for cancer-related cases [14], serving as a proxy for environmental fungal burden (*r *= 0.78, *p* = 0.01; Fig. 3C). In Japan, BA cases reported by the Biliary Atresia Registry [26] over 25 years strongly correlated with annual autopsy reports of visceral *Aspergillosis* [15, 16] (*r* = 0.85, *p* = 0.01; Fig. 3D), reflecting the community burden of *Aspergillus*. In contrast, no significant correlations were found with visceral *Candidiasis* (*r* = 0.54, *p* = 0.17; Fig. 3E), *Cryptococcus* (*r* = 0.42, *p* = 0.30; Fig. 3F), or *Mucormycetes* (*r* = 0.41, *p* = 0.31; Fig. 3G). For all correlations, the figures display both the 95% confidence intervals for Pearson’s *r* and shaded 95% confidence bands around the regression lines. These data underscore the distinct association of *Aspergillus* spp. in BA incidence.

## Discussion

Inspired by the clinical response of the index cholangitis case to antifungal agents (Fig. 1), we found evidence for a previously unrecognized association between *Aspergillus* spp. and fecal samples from BA patients. This was supported by 18S-based sequencing of our own samples (Table 1) and taxonomic analyses of shotgun metagenomes from an independent dataset (Fig. 2). Epidemiological data from the UK, Taiwan, and Japan further reinforced this association (Fig. 3). These observations are consistent with proteomic data suggesting host–fungal interactions, as BA feces were reported to contain ~347-fold higher levels of CHI3L1 [27], a protein that binds fungal chitin and protects against allergic asthma induced by *Aspergillus fumigatus* [28].

Careful examination of the index case suggests that the initial gastroenteritis phase responded to IV fluconazole, whereas recovery from the subsequent cholangitis phase required oral fluconazole. This raises the possibility of a two-hit sequence, in which an early immune priming phase is followed by aberrant immune hypersensitivity, accompanied by eosinophilia, after exposure to *Aspergillus* spp. and/or fungal-derived molecules in the gastrointestinal lumen. Such a framework could help explain several clinical features of BA. The priming phase may occur prenatally or perinatally and may be more common in infants of mothers with preexisting diabetes [29]. The second hit may be triggered by viral infection [30] or by environments with persistent *Aspergillus* exposure, such as neonatal intensive care units [31]. This stage could develop only after fungi have had sufficient opportunity to populate the gastrointestinal lumen, potentially facilitated by ciliary dysfunction [32]. Thus, *Aspergillus* spp. may provide a plausible mechanistic link for BA occurrence, although confirmatory studies are needed to establish causality.

Song et al. [22] employed SAOPaligner [33] to align reads against known microbial genomes, a method that may be less sensitive than Kraken 2 [12], which we used for fungal detection. Moreover, the number of available genomes in 2021, when their dataset was published, was likely limited. While k-mer–based tools such as Kraken 2 can yield false positives, the consistent detection of *Aspergillus* in both forward and reverse reads, with nearly identical abundance profiles (Fig. 2), strengthens the evidence for an association. A recent report [34] also highlighted the importance of *Bifidobacterium longum* in preserving native liver function in BA patients following Kasai portoenterostomy. Because many *Bifidobacterium* strains antagonize *Aspergillus* spp. [35], the presence of *B. longum* in BA cases may contribute to preserving native liver by limiting the impact of *Aspergillus* spp.

## Conclusion

In summary, our findings suggest an association between *Aspergillus* spp. and BA, supported by fecal sequencing, re-analysis of independent metagenomic data, and cross-country epidemiological correlations. These exploratory results are limited by small sample size, retrospective design, and lack of mechanistic validation, and should therefore be considered hypothesis-generating. Prospective and mechanistic studies will be required to confirm and extend these observations.

## Supplementary Information

Additional file 1: Supplementary Methods, Figure S1A-E, Figure S2A-E

## Data Availability

Sanger sequencing data are available from the corresponding author upon reasonable request. Fecal metagenome sequences and correlation datasets are available from previously published studies [13–16, 22–26].

## Declarations

### Generative AI and AI-assisted technologies in the writing process

During manuscript preparation, the authors used ChatGPT to enhance readability. After applying this tool, the authors reviewed and edited the content as needed and take full responsibility for the final manuscript.

## References

[1] Schwarz KB, Haber BH, Rosenthal P, Mack CL, Moore J, Bove K, et al. Extrahepatic anomalies in infants with biliary atresia: results of a large prospective North American multicenter study. Hepatology. 2013;58(5):1724–31. 23703680 10.1002/hep.26512 PMC3844083

[2] Kasai M. Treatment of biliary atresia with special reference to hepatic porto-enterostomy and its modifications. Prog Pediatr Surg. 1974;6:5–52. 4596366

[3] Yoeli D, Choudhury RA, Sundaram SS, Mack CL, Roach JP, Karrer FM, et al. Primary vs. salvage liver transplantation for biliary atresia: a retrospective cohort study. J Pediatr Surg. 2022;57(10):407–13. 35065808 10.1016/j.jpedsurg.2021.12.027

[4] Wu LN, Zhu ZJ, Sun LY. Genetic factors and their role in the pathogenesis of biliary atresia. Front Pediatr. 2022;10:912154. 35844731 10.3389/fped.2022.912154 PMC9277099

[5] Kotb MA, Kotb A, Talaat S, Shehata SM, El Dessouki N, ElHaddad AA, et al. Congenital aflatoxicosis, mal-detoxification genomics & ontogeny trigger immune-mediated Kotb disease biliary atresia variant: SANRA compliant review. Med (Baltimore). 2022;101(39):e30368. 10.1097/MD.0000000000030368 PMC9524989 36181129

[6] Riepenhoff-Talty M, Gouvea V, Evans MJ, Svensson L, Hoffenberg E, Sokol RJ, et al. Detection of group C rotavirus in infants with extrahepatic biliary atresia. J Infect Dis. 1996;174(1):8–15. 8656017 10.1093/infdis/174.1.8

[7] Tyler KL, Sokol RJ, Oberhaus SM, Le M, Karrer FM, Narkewicz MR, et al. Detection of reovirus RNA in hepatobiliary tissues from patients with extrahepatic biliary atresia and choledochal cysts. Hepatology. 1998;27(6):1475–82. 9620316 10.1002/hep.510270603

[8] Lorent K, Gong W, Koo KA, Waisbourd-Zinman O, Karjoo S, Zhao X, et al. Identification of a plant isoflavonoid that causes biliary atresia. Sci Transl Med. 2015;7(286):286ra67. 25947162 10.1126/scitranslmed.aaa1652 PMC4784984

[9] Altschul SF, Gish W, Miller W, Myers EW, Lipman DJ. Basic local alignment search tool. J Mol Biol. 1990;215(3):403–10. 2231712 10.1016/S0022-2836(05)80360-2

[10] Sayers EW, Bolton EE, Brister JR, Canese K, Chan J, Comeau DC, et al. Database resources of the national center for biotechnology information. Nucl Acid Res. 2022;50(D1):D20–6. 10.1093/nar/gkab1112 PMC8728269 34850941

[11] Waskom ML. Seaborn: statistical data visualization. J Open Source Softw. 2021;6(60):3021.

[12] Wood DE, Lu J, Langmead B. Improved metagenomic analysis with Kraken 2. Genome Biol. 2019;20(1):257. 31779668 10.1186/s13059-019-1891-0 PMC6883579

[13] Sung AH, Kiely G, Singh JK, Thomas S, Lough J, Smith M. Hospital-treated serious and invasive aspergillosis and candidiasis infections during the COVID-19 pandemic: a retrospective analysis of Hospital Episode Statistics data from England. BMJ Open. 2023;13(5):e070537. 37253500 10.1136/bmjopen-2022-070537 PMC10254895

[14] Hsiue HC, Wu TH, Chang TC, Hsiue YC, Huang YT, Lee PI, et al. Culture-positive invasive aspergillosis in a medical center in Taiwan, 2000–2009. Eur J Clin Microbiol Infect Dis. 2012;31(7):1319–26. 21997774 10.1007/s10096-011-1445-1

[15] Kume H, Yamazaki T, Togano T, Abe M, Tanuma H, Kawana S, et al. Epidemiology of visceral mycoses in autopsy cases in Japan: comparison of the data from 1989, 1993, 1997, 2001, 2005 and 2007 in annual of pathological autopsy cases in Japan. Med Mycol J. 2011;52(2):117–27. 21788723 10.3314/jjmm.52.117

[16] Suzuki Y, Togano T, Ohto H, Kume H. Visceral mycoses in autopsied cases in Japan from 1989 to 2013. Med Mycol J. 2018;59(4):E53-62. 30504616 10.3314/mmj.18-00003

[17] Virtanen P, Gommers R, Oliphant TE, Haberland M, Reddy T, Cournapeau D, et al. Scipy 1.0: fundamental algorithms for scientific computing in Python. Nat Methods. 2020;17(3):261–72. 32015543 10.1038/s41592-019-0686-2 PMC7056644

[18] Banos S, Lentendu G, Kopf A, Wubet T, Glockner FO, Reich M. A comprehensive fungi-specific 18S rRNA gene sequence primer toolkit suited for diverse research issues and sequencing platforms. BMC Microbiol. 2018;18(1):190. 30458701 10.1186/s12866-018-1331-4 PMC6247509

[19] Olsen GJ, Lane DJ, Giovannoni SJ, Pace NR, Stahl DA. Microbial ecology and evolution: a ribosomal RNA approach. Annu Rev Microbiol. 1986;40:337–65. 2430518 10.1146/annurev.mi.40.100186.002005

[20] Limon JJ, Skalski JH, Underhill DM. Commensal fungi in health and disease. Cell Host Microbe. 2017;22(2):156–65. 28799901 10.1016/j.chom.2017.07.002 PMC5573128

[21] Tsang CC, Tang JYM, Lau SKP, Woo PCY. Taxonomy and evolution of *Aspergillus*, *Penicillium* and *Talaromyces* in the omics era - past, present and future. Comput Struct Biotechnol J. 2018;16:197–210. 30002790 10.1016/j.csbj.2018.05.003 PMC6039702

[22] Song W, Sun LY, Zhu ZJ, Wei L, Qu W, Zeng ZG, et al. Characteristics of gut microbiota in children with biliary atresia after liver transplantation. Front Physiol. 2021;12:704313. 34262484 10.3389/fphys.2021.704313 PMC8273867

[23] Arshad A, Sutcliffe A, Jain V, Alizai N, Rajwal S, Kelly DA, et al. Reduced presentation of biliary atresia during the COVID-19 lockdown: a population based observational study. J Pediatr Gastroenterol Nutr. 2023;76(4):424–7. 36656748 10.1097/MPG.0000000000003706 PMC10012840

[24] Lin YC, Chang MH, Liao SF, Wu JF, Ni YH, Tiao MM, et al. Decreasing rate of biliary atresia in Taiwan: A survey, 2004–2009. Pediatrics. 2011;128(3):e530-6. 21873702 10.1542/peds.2011-0742

[25] Tiao MM, Tsai SS, Kuo HW, Chen CL, Yang CY. Epidemiological features of biliary atresia in Taiwan, a national study 1996–2003. J Gastroenterol Hepatol. 2008;23(1):62–6. 17725591 10.1111/j.1440-1746.2007.05114.x

[26] Nio M. Japanese biliary atresia registry. Pediatr Surg Int. 2017;33(12):1319–25. 29039049 10.1007/s00383-017-4160-x

[27] Watanabe E, Kawashima Y, Suda W, Kakihara T, Takazawa S, Nakajima D, et al. Discovery of candidate stool biomarker proteins for biliary atresia using proteome analysis by data-independent acquisition mass spectrometry. Proteomes. 2020. 10.3390/proteomes8040036. 33260872 10.3390/proteomes8040036 PMC7709124

[28] Mackel JJ, Garth JM, Jones M, Ellis DA, Blackburn JP, Yu Z, et al. Chitinase 3-like-1 protects airway function despite promoting type 2 inflammation during fungal-associated allergic airway inflammation. Am J Physiol Lung Cell Mol Physiol. 2021;320(4):L615–26. 33533316 10.1152/ajplung.00528.2020 PMC8238152

[29] Cavallo L, Kovar EM, Aqul A, McLoughlin L, Mittal NK, Rodriguez-Baez N, et al. The epidemiology of Biliary Atresia: Exploring the role of developmental factors on birth prevalence. J Pediatr. 2022;246(89–94):e2. 10.1016/j.jpeds.2022.03.038 PMC9332904 35364097

[30] Zuo T, Zhan H, Zhang F, Liu Q, Tso EYK, Lui GCY, et al. Alterations in fecal fungal microbiome of patients with covid-19 during time of hospitalization until discharge. Gastroenterol. 2020;159(1302–10):e5. 10.1053/j.gastro.2020.06.048 PMC7318920 32598884

[31] Mohammad N, Huguenin A, Lefebvre A, Menvielle L, Toubas D, Ranque S, et al. Nosocomial transmission of *Aspergillus flavus* in a neonatal intensive care unit: long-term persistence in environment and interest of MALDI-ToF mass-spectrometry coupled with convolutional neural network for rapid clone recognition. Med Mycol. 2024. 10.1093/mmy/myad136. 38142226 10.1093/mmy/myad136

[32] Lam WY, Tang CS, So MT, Yue H, Hsu JS, Chung PH, et al. Identification of a wide spectrum of ciliary gene mutations in nonsyndromic biliary atresia patients implicates ciliary dysfunction as a novel disease mechanism. eBioMedicine. 2021;71:103530. 34455394 10.1016/j.ebiom.2021.103530 PMC8403738

[33] Li R, Yu C, Li Y, Lam TW, Yiu SM, Kristiansen K, et al. SOAP2: an improved ultrafast tool for short read alignment. Bioinformatics. 2009;25(15):1966–7. 19497933 10.1093/bioinformatics/btp336

[34] Lee CS, Lin CR, Chua HH, Wu JF, Chang KC, Ni YH, et al. Gut *Bifidobacterium longum* is associated with better native liver survival in patients with biliary atresia. JHEP Rep. 2024;6(7):101090. 39006502 10.1016/j.jhepr.2024.101090 PMC11246047

[35] Ghazvini RD, Kouhsari E, Zibafar E, Hashemi SJ, Amini A, Niknejad F. Antifungal activity and aflatoxin degradation of *Bifidobacterium bifidum* and *Lactobacillus fermentum* against toxigenic *Aspergillus parasiticus*. Open Microbiol J. 2016;10:197–201. 28077976 10.2174/1874285801610010197 PMC5204065

