# Supplementary material for: Cross-country multi-modal evidence links Aspergillus to biliary atresia
Source: Gut Pathog. 2025 Nov 17;17:94. doi: 10.1186/s13099-025-00772-7 (PMC12621361; doi:10.1186/s13099-025-00772-7)
Supplement: Supplementary file 1 — Additional file 1: Supplementary Methods, Figure S1A-E, Figure S2A-E [file 13099_2025_772_MOESM1_ESM.docx]

**Supplementary Methods**

**Fecal DNA extraction**

Stool samples were preserved at −80 °C until processing. For the index case, DNA was extracted from 50–200 mg of stool using the ZymoBIOMICS DNA Miniprep Kit (Zymo Research, Cat. D4300) according to the manufacturer’s protocol. For the broader case–control analysis, fecal DNA from BA patients and controls was extracted using the QIAamp PowerFecal Pro DNA Kit (QIAGEN, Hilden, Germany).

**18S PCR and Sanger sequencing**

Polymerase chain reactions (PCR) were performed using the KAPA HiFi HotStart ReadyMix PCR Kit (Roche, Cat. KK2602). To amplify the 18S rRNA V1–V8 region (1, 2), DNA templates were subjected to PCR with primers 18S-F-bc1005C-P5 (5′-AATGATACGGCGACCACCGAGATCTACACGTGAGCTGAGAGCGCACCATGCATGTCTAAGTWTAA-3′) and 18S-R-bc1033C-P7 (5′-CAAGCAGAAGACGGCATACGAGATTCTCTGACGCTGCTCTAICCATTCAATCGGTAIT-3′). Reactions were run on a MiniAmp Plus Thermal Cycler (Thermo Fisher Scientific, Cat. A37835) under the following conditions: initial denaturation at 95 °C for 3 min, followed by 5 cycles of 98 °C for 30 s, 54 °C for 30 s, and 72 °C for 90 s; 5 cycles of 98 °C for 30 s, 51 °C for 30 s, and 72 °C for 90 s; 30 cycles of 98 °C for 30 s, 48 °C for 30 s, and 72 °C for 90 s; and a final extension at 72 °C for 3 min 30 s. Yeast cDNA and ddH₂O were used as positive and negative controls, respectively.

For semi-nested amplification of the V7–V8 or V4–V8 regions, an initial V1–V8 PCR was performed as above, except that the final 30 cycles were reduced to 20. One microliter of the resulting amplicon was then used as the template for the second-round PCR. The V7–V8 region was amplified with primers 18S-F-nu-SSU-1333-5′ (5′-CGWTAACGAACGAGACCT-3′) and 18S-R-bc1033C-P7. The V4–V8 region was amplified with primers 18S-V4-F (5′-CCAGCAGCCGCGGTAATTCC-3′) and 18S-R-bc1033C-P7. Thermocycling conditions for both semi-nested PCRs were as follows: 95 °C for 3 min; 5 cycles of 98 °C for 30 s, 54 °C for 30 s, and 72 °C for 1 min; 5 cycles of 98 °C for 30 s, 51 °C for 30 s, and 72 °C for 1 min; 20 cycles of 98 °C for 30 s, 48 °C for 30 s, and 72 °C for 1 min; and a final extension at 72 °C for 3 min 30 s. Semi-nested amplicons were purified using the MinElute PCR Purification Kit (QIAGEN, Cat. 28006) before downstream applications such as Sanger sequencing or TA cloning.

**TA cloning**

PCR products from semi-nested 18S V4–V8 amplification were prepared for cloning by A-tailing with FastStart Taq DNA Polymerase (Roche, Cat. 12161508103). The enzyme was activated at 95 °C for 4 min, followed by incubation at 72 °C for 10 min to add 3′-adenine overhangs. A-tailed amplicons were ligated into the pGEM®-T Easy Vector (Promega, Cat. A1360) according to the manufacturer’s instructions.

Ligation mixtures were directly transformed into *E. coli* DH5α competent cells (Yeastern Biotech, ECOS™ 101). Transformed cells were plated on LB agar containing 100 μg/mL ampicillin, IPTG, and X-Gal, then incubated overnight at 37 °C for blue–white screening. White colonies were selected, cultured, and subjected to plasmid DNA extraction using the FAVORGEN Plasmid DNA Extraction Kit (Cat. FAPDE300). Extracted plasmids were digested with EcoRI (New England Biolabs, Cat. R3101S) at 37 °C for 1 h to confirm insert size. Plasmids containing appropriately sized inserts were submitted for Sanger sequencing.

**16S PCR**

Bacterial 16S rRNA genes were amplified with the KAPA HiFi HotStart ReadyMix PCR Kit (Roche, Cat. KK2602), following published protocols (2). The full V1–V9 region was targeted using primers 16S-F-bc1005-P5 (5′-AATGATACGGCGACCACCGAGATCTACACCACTCGACTCTCGCGTAGRGTTYGATYMTGGCTCAG-3′) and 16S-R-bc1033-P7 (5′-CAAGCAGAAGACGGCATACGAGATAGAGACTGCGACGAGARGYTACCTTGTTACGACTT-3′). PCR amplification was performed using the following cycling program: an initial denaturation at 95 °C for 3 min; 27 cycles of 95 °C for 30 s, 57 °C for 30 s, and 72 °C for 1 min; and a final extension at 72 °C for 3 min. *E. coli* DH5α genomic DNA and nuclease-free water served as positive and negative controls, respectively.

**Supplementary Figures**

**
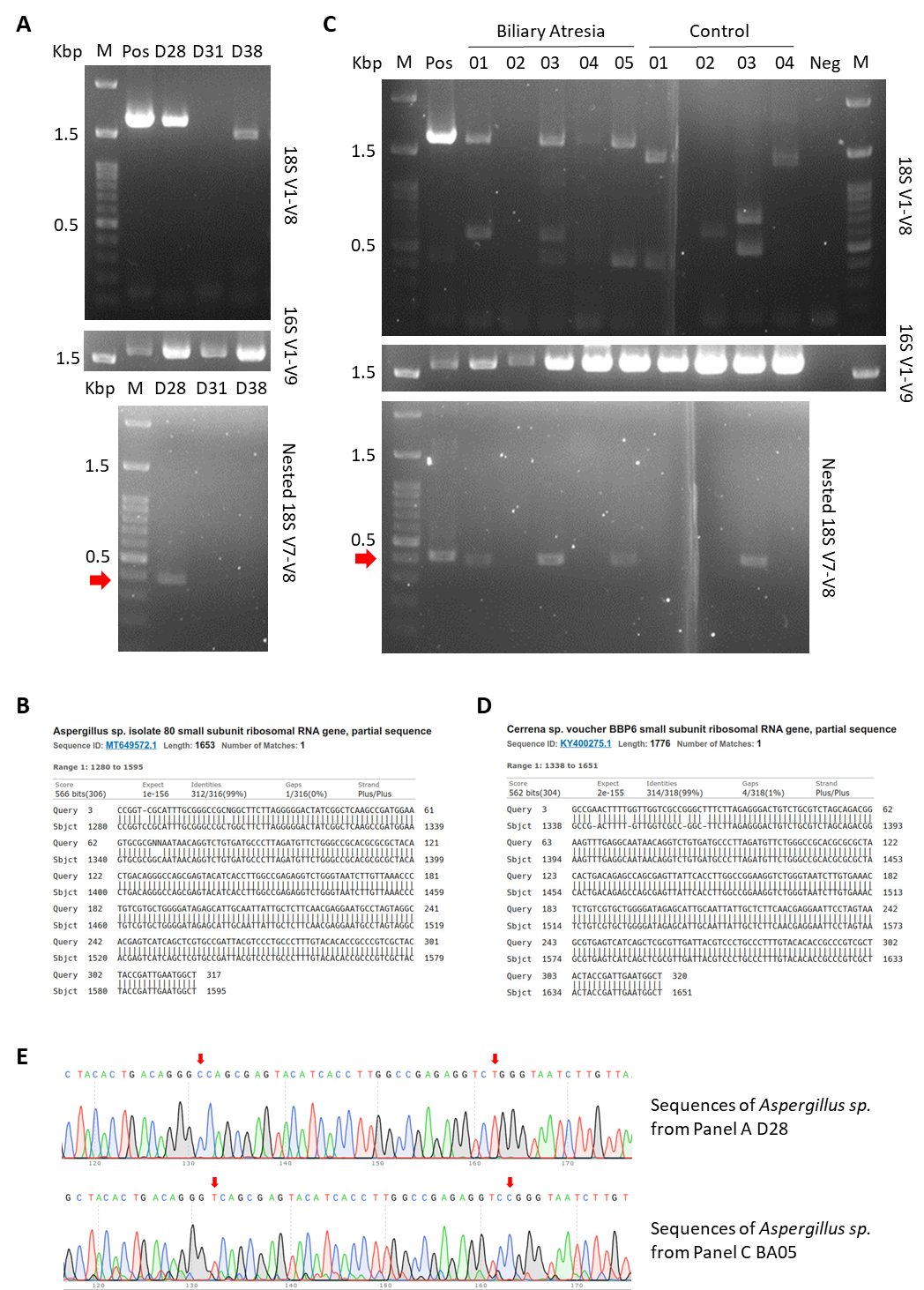
**

**Figure S1.** Molecular identification of fungal species using 18S ribosomal sequences. **A** PCR amplification of the 18S V1–V8 region and semi-nested V7–V8 region (red arrow) from fecal DNA of the cholangitis case. 16S V1–V9 PCR served as an internal control. **B** *Aspergillus* sp. was identified in stool from the index case. **C** 18S V1–V8 and semi-nested V7–V8 (red arrow) PCRs performed on fecal DNA from BA patients and healthy controls. **D** V7–V8 sequences from BA01 matched *Cerrena* sp. **E** *Aspergillus* spp. sequences differed between the cholangitis case and BA05.

**
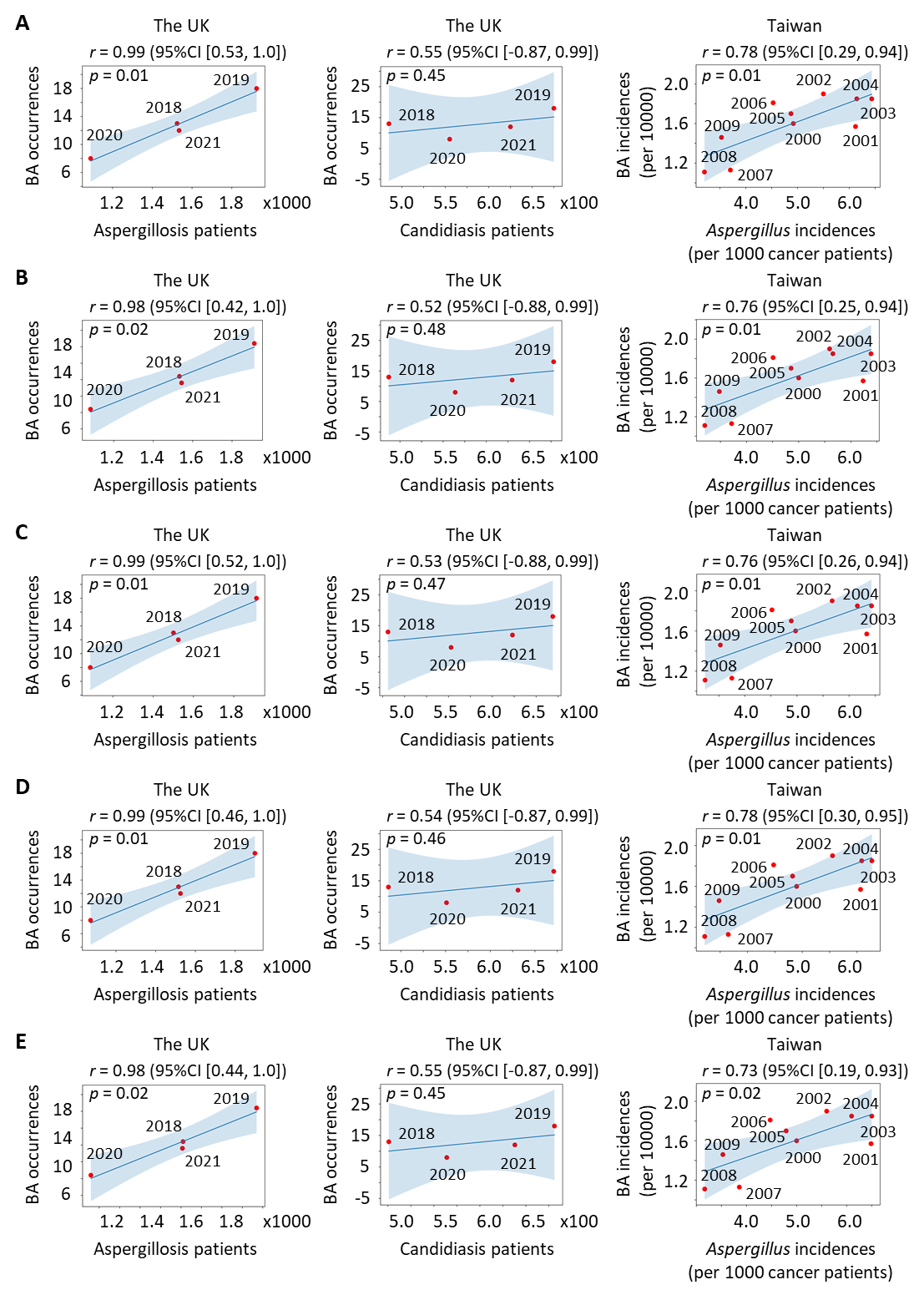
**

**Figure S2.** Independent manual measurements of epidemiological correlations.
To assess reproducibility, correlations between BA incidence and *Aspergillus* spp. burdens in the UK and Taiwan were independently re-measured by five additional individuals, including three non-authors. The resulting analyses (A–E) reproduced the patterns shown in Figure 3A–C, demonstrating high consistency across raters.

**References**

1. Banos S, Lentendu G, Kopf A, Wubet T, Glockner FO, Reich M. A comprehensive fungi-specific 18S rRNA gene sequence primer toolkit suited for diverse research issues and sequencing platforms. BMC Microbiol. 2018;18(1):190.

2. Olsen GJ, Lane DJ, Giovannoni SJ, Pace NR, Stahl DA. Microbial ecology and evolution: a ribosomal RNA approach. Annu Rev Microbiol. 1986;40:337-65.
